# Evaluation of the impact of universal testing for gestational diabetes mellitus on maternal and neonatal health outcomes: a retrospective analysis

**DOI:** 10.1186/1471-2393-14-317

**Published:** 2014-09-09

**Authors:** Diane Farrar, Lesley Fairley, John Wright, Derek Tuffnell, Donald Whitelaw, Debbie A Lawlor

**Affiliations:** Bradford Institute for Health Research, Bradford Royal Infirmary, Bradford, UK; MRC Integrative Epidemiology Unit at the University of Bristol, Bristol, UK; Women’s and Newborn Unit, Bradford Royal Infirmary, Bradford, UK

**Keywords:** Gestational diabetes, Universal and selective screening, Risk factors, Oral glucose tolerance test, Perinatal outcomes

## Abstract

**Background:**

Gestational diabetes (GDM) affects a substantial proportion of women in pregnancy and is associated with increased risk of adverse perinatal and long term outcomes. Treatment seems to improve perinatal outcomes, the relative effectiveness of different strategies for identifying women with GDM however is less clear.

This paper describes an evaluation of the impact of a change in policy from selective risk factor based offering, to universal offering of an oral glucose tolerance test (OGTT) to identify women with GDM on maternal and neonatal outcomes.

**Methods:**

Retrospective six year analysis of 35,674 births at the Women’s and Newborn unit, Bradford Royal Infirmary, United Kingdom.

**Results:**

The proportion of the whole obstetric population diagnosed with GDM increased almost fourfold following universal offering of an OGTT compared to selective offering of an OGTT; Rate Ratio (RR) 3.75 (95% CI 3.28 to 4.29), the proportion identified with severe hyperglycaemia doubled following the policy change; 1.96 (1.50 to 2.58). The case detection rate however, for GDM in the whole population and severe hyperglycaemia in those with GDM reduced by 50-60%; 0.40 (0.35 to 0.46) and 0.51 (0.39 to 0.67) respectively. Universally offering an OGTT was associated with an increased induction of labour rate in the whole obstetric population and in women with GDM; 1.43 (1.35 to 1.50) and 1.21 (1.00 to1.49) respectively. Caesarean section, macrosomia and perinatal mortality rates in the whole population were similar. For women with GDM, rate of caesarean section; 0.70 (0.57 to 0.87), macrosomia; 0.22 (0.15 to 0.34) and perinatal mortality 0.12 (0.03 to 0.46) decreased following the policy change.

**Conclusions:**

Universally offering an OGTT was associated with increased identification of women with GDM and severe hyperglycaemia and with neonatal benefits for those with GDM. There was no evidence of benefit or adverse effects in neonatal outcomes in the whole obstetric population.

**Electronic supplementary material:**

The online version of this article (doi:10.1186/1471-2393-14-317) contains supplementary material, which is available to authorized users.

## Background

Gestational diabetes mellitus (GDM) affects 2-6% of pregnant women and is associated with increased risk of important adverse perinatal outcomes, including macrosomia and birth injury [1, 2]. There is also evidence of increased long term risk of type 2 diabetes [3] and consequent cardiovascular disease in the mothers [4] and possibly of increased long term risk of obesity and associated adverse cardio-metabolic risk in offspring [5–8].

Evidence is increasing that treatment of GDM improves perinatal outcomes [9] supporting the case for improved identification of women with GDM. There is debate however about the relative effectiveness of different strategies for identifying women with GDM, largely because of the lack of good quality evidence. This has led to variation in clinical guidelines and practice for detecting GDM between, and within, countries. Strategies include: case by case assessment [10], selective (75 g or 100 g) oral glucose tolerance testing of high risk women identified using specific risk factors or a 50 g glucose challenge test [11] and universal testing, i.e. offering all women an oral glucose tolerance test (OGTT) [12].

To date in the United Kingdom (UK) there has been no recommendation to offer all women an OGTT, in practice, and more recently in clinical guidelines, selective testing of high risk women has been undertaken. Prior to 2008 in the UK there was no national recommended screening strategy to identify women with GDM. Screening if it was conducted, was at the discretion of the clinician and based on variable use of risk factors [13–15]. When risk factor screening was undertaken, a two-step approach was preferred: clinicians made a clinical assessment of each woman’s risk and offered a two hour 75 g OGTT to identify GDM, with a diagnosis based on the World Health Organisation (WHO) criteria [16].

Since 2008 UK national clinical guidance has recommended that all women are screened by assessment of specific risk factors at their first pregnancy appointment. Any pregnant woman (not previously identified as having type 2 diabetes) with one or more risk factor: family history of diabetes; South Asian; black or middle eastern ethnicity; previous history of having a baby with macrosomia; or body mass index (kg/m^2^) (BMI) ≥30, should be offered an OGTT between 24 and 28 weeks gestation [11].

The American College of Obstetricians and Gynecologists (ACOG) also recommends a two-step approach. Women are screened for GDM at 24 to 26 weeks, either by patient history, risk factors or 50 g one hour glucose challenge test and if screen positive offered a 100 g OGTT. GDM diagnosis is made using criteria from Carpenter and Coustan or the National Diabetes Data Group [17]. By contrast the International Association of Diabetes in Pregnancy Study Group [12] (indorsed by the American Diabetes Association) recommend all women not previously identified as having type 2 diabetes (irrespective of risk factors) are offered a diagnostic 75 g OGTT at 24–28 weeks. GDM is diagnosed if any one of plasma glucose levels are >5.0 mmol/l, one hour >9.9 mmol/l or two hour >8.4 mmol/l is found [18].

Current screening recommendations are based on evidence from observational studies with no evidence that diagnosis using any of the above strategies improves perinatal or long term adverse outcomes or is cost-effective. Consequently the US Preventative Services Taskforce recommends clinicians discuss screening for GDM with each woman and case by case decisions made based on risk status [10]. Selectively offering women an OGTT based on risk factor assessment and universally offering an OGTT to some extent represent the extremes of possible approaches for identifying women at risk. Theoretically the former approach may have a high false negative rate and miss the opportunity to prevent adverse perinatal outcomes in women at risk, whereas the latter approach may over-diagnose [19], cause unnecessary anxiety [20] and may not be cost-effective in improving perinatal outcomes. Recent policy changes from a clinician led risk factor screening approach to universal offering a diagnostic 75 g OGTT in Bradford provided us with the opportunity to compare these two approaches.

### Setting

Bradford is a city in the North of England with high levels of deprivation. Approximately half of births are to women of South Asian origin and a fifth of White British pregnant women are obese (unpublished routine study hospital data from 2012). In response to the Bradford District Mortality Commission, which was undertaken in 2006 and highlighted rising infant mortality and the poor health of pregnant women in the city [21], a number of changes to clinical practice were implemented, including offering a diagnostic OGTT to all pregnant women at 26–28 weeks gestation. Prior to 2007 women were offered an OGTT at 26–28 weeks gestation following individual assessment of risk status. The aim of this study was to evaluate the policy change from case by case risk factor based assessment and selective testing to universal testing (offering a diagnostic OGTT to all women) to identify gestational diabetes.

## Methods

### Participants

We conducted a before and after comparison using data from all women who gave birth at the Bradford Royal Infirmary between 2004 to 2006 (before group) and 2008 to 2010 (after group). Those who gave birth in 2007 were excluded as this year contained a mixture of women selectively offered an OGTT and women universally offered an OGTT. In the UK, anonymised routinely collected data of those accessing National Health Service care may be used to evaluate service provision. Those using such data for service evaluation are not required to obtain verbal or written consent from patients or obtain ethical approval. Therefore neither patient consent nor ethical approval for this study was obtained, as routinely collected hospital data were used to evaluate service provision. Analyses were conducted on a completely anonymised dataset and none of the authors had access to any patient identifiers [22].

### Oral glucose tolerance test

The 75 g OGTT was administered during the study period between 26–28 weeks gestation. Fasting plasma glucose and two hour post-load plasma glucose measurements were obtained. Criteria used to diagnose GDM was based on WHO recommendations (i.e. either fasting glucose ≥6.1 mmol/L or two-hour glucose ≥7.8 mmol/L) and did not change throughout the study period [16].

### Extraction of data

Data were extracted from hospital written and electronic medical records. Maternal risk factors (previous GDM, family history of diabetes, previous history of giving birth to a baby with macrosomia, obesity, South Asian or black ethnicity, glycosuria, raised result of any random blood glucose test and increased liquor volume) were available for women who had completed an OGTT between 2004 to 2006 (case by case assessment and selectively tested group). These risk factors were not available for women during this time period that did not complete an OGTT, nor were they available for women who were pregnant after 2007, other than ethnicity which was available for all women. Data on whether an OGTT was performed, whether they were diagnosed with GDM and maternal and neonatal outcomes (induction of labour, caesarean birth, macrosomia (birth weight equal to or greater than 4 kg), perinatal mortality, admission to neonatal unit, Erb’s palsy, fractured clavicle, were available for all births (before and after the policy change) and included in the analyses.

Data on risk factors (where available), completion of the OGTT and OGTT results were abstracted from paper medical or electronic records, outcome data were provided by the hospital electronic records system for the period 2004 to 2006. Neonatal transitional care unit data and data for 2008 to 2010 were abstracted from paper medical or electronic records. Research midwives abstracted data from paper records using a pre-prepared coded data extraction sheet. A 5% random sample of these data were independently abstracted by a research fellow; for all field codes there was greater than 98% agreement between the research fellow and research midwife abstractions. Electronic data were transferred from the electronic medical record to a research database and merged with the abstracted paper record data. These electronic data were manually checked against stored paper records and again high levels (98%) of agreement were found. We also compared the medical records of GDM diagnoses with the biochemical laboratory records of test results and found very high levels of agreement (>99%).

### Analysis

All analyses were conducted using Stata version 10 [23]. We calculated the percentage of women with an identified risk factor and used the binomial distribution to calculate 95% confidence intervals for these prevalence’s. We used the same method to calculate the percentage and 95% confidence intervals for women who had an OGTT between 2008 and 2010 (i.e. period during which there was a policy of universal OGTT testing).

The proportion of women with GDM, either a fasting blood glucose ≥6.1 mmol/L, or a two hour blood glucose ≥7.8 mmol/L or both and of these the proportion with severe hyperglycaemia, either a fasting blood glucose ≥7 mmol/L, or a two hour blood glucose ≥11.1 mmol/L or both was estimated. The proportion of women/infants with each risk factor (where available) and outcome (diagnosis of GDM, induction of labour, caesarean birth, macrosomia, perinatal mortality, admission to neonatal unit, Erb’s palsy and fractured clavicle) was estimated for each time period. Rate ratios (RR), comparing outcomes after the introduction of the universal offer of a diagnostic OGTT, to those before this policy, together with their 95% confidence intervals were estimated using the epitab command in Stata [23].

## Results

Between 2004 and 2006 (selectively offered group), 17,160 women gave birth; 1162 (7%) were offered an OGTT following risk assessment by their clinician and 1151 completed the test (7% of all women and 99% of those offered the test). Between 2008 and 2010, (universally offered group) 18,514 women gave birth, 18,328 (99%) were offered an OGTT and 11,516 completed the test (62% of all women and 63% of those offered the test) (Table 1). For births occurring between 2004–2006, amongst those who completed the OGTT 58% were from an ethnic group with increased risk (the most common risk factor) compared with 55% after the policy change (risk ratio 0.95, 95% CI: 0.87 to 1.03). Obesity was the second most common individual risk factor for women selectively offered an OGTT (Additional file 1: Table S1). A small proportion (1%) of the women in the universally offered group were not offered an OGTT, reasons were: pre-existing diabetes or late attendance for antenatal care. Of those attending for the OGTT (after group only), 3% did not complete the test either because they had been unable to fast overnight or were unable to drink the glucose solution due to nausea. These women were all offered a second appointment and over 99% completed the test at that second appointment.

**Table 1:** **Association between selective and universal offer of an oral glucose tolerance test (OGTT) with gestational diabetes and severe hyperglycaemia detection rate (number tested with percentage of whole population and percentage of screened population in parenthesis)**

	Selective offering of an OGTT				Universal offering of an OGTT				
	2004	2005	2006	2004-2006	2008	2009	2010	2008-2010	Rate ratio comparing detection rates after to before (95% CI) for the whole population and for the screened population
Whole population	5512	5784	5864	17160	6162	6251	6101	18514	
Completed OGTT	323 (6)	299 (5)	529 (9)	1151 (7)	3797 (62)	3947 (63)	3772 (62)	11516 (62)	
GDM^a^ identified	62 (1, 19)	83 (1, 28)	135 (2, 26)	280 (2, 24)	311 (5, 8)	398 (6, 10)	423 (7, 11)	1132 (6, 10)	3.75 (3.28 - 4.29) 0.40 (0.35 - 0.46)
Severe hyperglycaemia^b^ identified	21 (0.4, 34)	33 (0.6, 40)	29 (0.5, 21)	83 (0.5, 30)	46 (0.7, 14)	63 (1.0, 16)	63 (1.0, 16)	172 (1.0, 15)	1.96 (1.50 - 2.58) 0.51 (0.39 - 0.67)

OGTT = oral glucose tolerance test.

GDM = gestational diabetes mellitus, CI = confidence interval.

^a^GDM = either a fasting blood glucose ≥6.1 mmol/L, or a two hour blood glucose ≥7.8 mol/L or both.

^b^Severe hyperglycaemia = either a fasting blood glucose ≥7 mmol/L, or a two hour blood glucose ≥11.1 mmol/L or both.

The proportion diagnosed with GDM increased almost fourfold after the introduction of the policy of offering a diagnostic OGTT to all pregnant women compared to selectively offering the test, (2% to 6%) Rate Ratio (RR) (3.75 95% CI 3.28 to 4.29) (Table 1). The proportion identified as having severe hyperglycaemia doubled following the change in policy (0.5% to 1.0%, 1.96 1.50 to 2.58) (Table 1). However, the population case detection rate (for both GDM and severe hyperglycaemia in those with GDM) reduced by 50-60% following universal offering of a diagnostic OGTT, reflecting an increase in those offered the test who were not at risk (Table 1).

Table 2 shows the testing strategies comparison of adverse maternal and neonatal outcomes. Induction of labour rate increased both in the whole obstetric population and in women with GDM after the introduction of offering universal OGTT. The caesarean section rate in the whole population was similar before and after the introduction of offering universal OGTT, but introduction of this universal offer was associated with a reduction in caesarean section rates amongst those with GDM. Similarly, offering universal OGTT was not associated with a change in the rate of macrosomia in the whole obstetric population, but was associated with a marked reduction in its rate amongst those with GDM. There was also a reduction in perinatal mortality for women with GDM after the introduction of the policy and some evidence of a weaker reduction in the whole population, but the latter rate ratio had wide confidence intervals that included the null value.

**Table 2:** **Association between selective and universal offer of an OGTT to identify gestational diabetes and risk of adverse maternal and neonatal outcomes in the whole obstetric population and in women with gestational diabetes**

	Whole obstetric population			Women identified with gestational diabetes		
	Selective offer of an OGTT 2004–2006 N = 17160 N (%)	Universal offer of an OGTT 2008–2010 N = 18514 N (%)	Rate ratio comparing after to before (95% CI)	Selective offer of an OGTT 2004–2006 N = 280 N (%)	Universal offer of an OGTT 2008–2010 N = 1132 N (%)	Rate ratio comparing after to before (95% CI)
Induction of labour	2422 (14.1)	3678 (20.0)	1.43 (1.35- 1.50)	120 (42.9)	587 (51.9)	1.21 (1.00-1.49)
Caesarean birth	3477 (20.3)	3709 (20.0)	1.00 (0.96-1.05)	122 (43.6)	345 (30.5)	0.70 (0.57-0.87)
Macrosomia (birth weight ≥4 kg)	1105 (6.4)	1219 (6.6)	1.04 (0.95-1.12)	45 (16.1)	50 (4.4)	0.22 (0.15-0.34)
Perinatal mortality	228 (1.3)	212 (1.1)	0.86 (0.71-1.04)	4 (1.4)	8 (0.7)	0.12 (0.03-0.46)
Admitted to NNU	1670 (9.7)	1497 (8.1)	0.83 (0.77-0.89)	69 (24.6)	118 (10.4)	0.42 (0.31-0.58)
Erb’s palsy	7 (0.04)	12 (0.06)	1.59 (0.58-4.76)	*	*	*
Fractured clavicle	4 (0.02)	6 (0.03)	1.39 (0.33-6.70)	*	*	*

OGTT = oral glucose tolerance test.

NNU: neonatal unit; *insufficient numbers to perform analyses for these outcomes in those identified with gestational diabetes.

Offering universal OGTT was also associated with a reduced rate of admission to the neonatal unit for the whole obstetric population and amongst those with GDM. The rates of Erb’s palsy and fractured clavicle in the infants appeared similar before and after the policy change, but at both time points rates of these outcomes were very low and the ratio estimates have very wide confidence intervals that include any association from a marked reduction to a marked increase in risk.

## Discussion

Changing antenatal care policy to identify women with GDM from case by case assessment and selective diagnostic OGTT, to offering a diagnostic OGTT to all pregnant women was associated with increased rates of GDM diagnoses and severe hyperglycaemia in the whole obstetric population, but with lower detection rates in those who completed the OGTT. This suggests universal offer of OGTT is associated with the increased identification of mild-moderate hyperglycaemia. There was also a reduction from 94% to 63% of those offered a diagnostic OGTT who accepted and completed the test. Induction of labour rate in the whole obstetric population and in those diagnosed with GDM increased when all women were offered a universal diagnostic OGTT, with no overall increase in caesarean section rate and a decrease in those with GDM. Offering all women diagnostic testing was also associated with improved neonatal outcomes in women identified with GDM, including a marked reduction in rates of macrosomia, neonatal mortality and admissions to neonatal care.

There was no strong evidence that offering an OGTT to all women affected most outcome rates for the whole obstetric population. There was some suggestion that perinatal mortality may have reduced in the whole obstetric population following the policy change, but here the confidence intervals were wide and just included the null value.

Thus, overall these results suggest that universal testing with OGTT in a high risk population such as that in Bradford with a large proportion at risk because of their ethnicity or obesity has some beneficial effects in terms of reducing adverse perinatal outcomes in those identified with GDM, without increasing caesarean section rates or adverse neonatal outcomes in the whole obstetric population. There were no changes to treatment policy during the study period that could account for the differences in outcomes demonstrated. A ‘step up’ approach was followed depending on severity of hyperglycaemia, whereby diet and exercise modification was advised and metformin and/or insulin added accordingly.

The reduced uptake of the invitation for a universally offered OGTT is similar to other studies who have reported rates of 58% [24] and 73% [25] and may reflect a difference in attitudes to a test once it is offered to everyone. It is plausible that both health care practitioners and the women themselves see the test as less important when it is not restricted to those designated as high risk following individual assessment. For example, in the absence of risk factors health professionals may place less emphasise on the importance of the test. Unfortunately, we did not have detailed information on risk factors in both those attending antenatal care before and after the change in policy to examine whether uptake amongst the most at risk had changed over time. It is also possible that those who declined the test were different in some way to those who accepted the test offer and that any benefits of universal testing may be greater if uptake could be improved from 63%, though we are unable to examine this here. Uptake of OGTT offer however may increase over time as clinicians and women become more familiar with the test and reasons for testing.

The potential benefits of offering universal testing and treatment of cases must be weighed against the cost of this service and any possible adverse effects to the pregnant women. Criteria for GDM diagnosis did not change across the study period, however it is notable that before the introduction of universal diagnostic testing three women were tested for each woman diagnosed with GDM, whereas once universal invitation was introduced ten women were tested for each woman diagnosed. If it were possible to increase the uptake of testing with universal invitation this difference would likely increase.

The small proportion at both time points of women who presented for the test, but could not complete it, because they had not fasted or were nauseous following drinking the glucose solution suggests that once a woman has decided to accept the invitation the test is feasible in the vast majority. We are unable in this study to examine cost-effectiveness as we do not have comprehensive data on the extent of risk factor screening that was completed in all women in the before group and since this is a before and after comparison we are only able to assess association rather than causation/effect.

There were no changes to criteria thresholds for GDM diagnosis within the study period, which would be likely to affect prevalence of GDM, [26] even so prevalence increased irrespective of strategy used. The increasing prevalence of maternal obesity and an increasing awareness amongst clinicians of the association between obesity and poor pregnancy outcomes may be responsible for the changes seen. For example, in 2004 three women were tested for GDM because of a raised BMI, in 2005, 17 women and in 2006, 225 women. Different risk factors are associated with different degrees of risk for GDM development. South Asian ethnicity and family history of diabetes carry greater risk of GDM development compared with BMI ≥30 [27]. However, we found no difference in the proportion of women who completed the OGTT before and after introduction of universal testing who belonged to an at-risk ethnic group, which is perhaps surprising as 50% of the study population are of South Asian ethnicity, this may reflect a belief that the use of ethnicity as a risk factor would result in too great a cost burden at the study hospital prior to the policy change or that the risk conveyed was not great enough to necessitate screening.

Our findings with respect to associations in the group of women who are diagnosed with GDM are consistent with randomised trials of more intensive treatment of GDM, suggesting that the increased detection rates did result in effective treatment in those with GDM. For example, recent randomised trials suggest that intensive treatment effectively reduces macrosomia and other adverse neonatal outcomes [28, 29]. With respect to maternal outcomes these trials had different findings, with one showing intensive treatment resulted in an increase in induction rates (as in our study), but no effect on caesarean section rates [28] and the second, no difference in induction rate and a reduction in caesarean section rates (the latter as in our study) [29].

The reduction in rates of macrosomia, comparing births after the introduction of universal offering of OGTT to those before is also consistent with substantial evidence that maternal hyperglycaemia causes increased birth size overall and increased adiposity at birth [8, 30]. Macrosomia complicates the intrapartum, rather than the antenatal period, and is therefore more likely to be associated with birth trauma and arrested labour rather than perinatal mortality [31]. In our study population the numbers experiencing birth trauma (Erb’s palsy or fractured clavicle) were too few to make meaningful conclusions about the change in policy to offering universal testing.

### Implications for research

Further research is needed to understand why, when all women were offered an OGTT only 63% attended for the test, compared with 99% when the OGTT was only offered to those identified as at risk. It is also important to investigate what the effect of increasing uptake of the test might be. Ultimately, randomised trials that compare universal testing and selective testing and assessment of a range of perinatal outcomes are required, but such trials would require a very large sample size. Continued evaluation of the Bradford population and other similar populations where there is a policy change provide some evidence of the likely impact of universal testing. It is also important to include assessment of the cost-effectiveness of strategies to identify and treat GDM, on-going work may help to answer this question [32].

### Strengths and limitations

A key strength of our study is its ability to examine the association of offering universal testing for OGTT compared with a more conservative strategy of selective testing based on individual assessment of risk. We were able to include in our analyses all women giving birth in Bradford over the study period examined and therefore results are unlikely to be affected by selection bias and the numbers included in the analyses are large. This approach provides a ‘real life’ evaluation of what happens when health care policy is changed, rather than findings from randomised trials that may overestimate the effect of an intervention [33, 34]. Whilst routine clinical data may be less accurate than data collected specifically for research purposes we carefully checked the reliability of data from different sources, where possible (e.g. electronic and paper medical records and laboratory results) and completed an independent abstraction of a random sample of data from medical records to improve the validity of the data we have used here.

The main weakness of this study is that it is a before and after comparison and therefore we cannot assume that any associations are caused by the change in policy of offering an OGTT to all pregnant women. For 2006 to 2008 we only had access to aggregated data for outcomes therefore we could only examine unadjusted associations and not adjust analyses to take account of the individual level background characteristics and confounders of the population and how these might have changed over time. Although there were no other changes in policy, any other characteristic that changed over this period could explain the findings we have observed (i.e. could have confounded our assumed association of change to universal offering of an OGTT with the outcomes assessed). For example, there was an increase in induction of labour rate following the introduction of universal offer of an OGTT, both for the whole obstetric population and for those with GDM, which cannot be wholly accounted for by the increase in GDM diagnosis.

As noted above there was already evidence that the rate of diagnosis of GDM was increasing prior to the change in policy, but the rate of this increase was much more marked after the policy was introduced. Despite the increasing rates of diagnosis before the policy change, there were still improved outcomes for the group universally offered an OGTT compared to the group selectively offered an OGTT.

Data on detailed risk factor assessment for all women across the whole period was not available; data were only available for those in the before group who completed an OGTT. Thus, we were unable to examine how well risk factor screening was implemented before the offer of universal testing or to compare risk factors between those who completed an OGTT and those who declined the invitation after the policy change. As noted above we are unable to complete a cost-effectiveness analysis in this study. Lastly, the population of Bradford are a high risk group as half of births are to women of South Asian origin and a fifth of the non-South Asian population are obese. Thus, at least 60% of this population would have at least one risk factor deemed by the NICE guidelines introduced in 2008, to make them eligible for an OGTT [11]. We cannot conclude that the results found in this population would necessarily generalise to other populations or to populations where other strategies or criteria are used, but given the whole population coverage it is likely that they would generalise to populations with similar high levels of risk.

## Conclusion

Our results suggest that offering all women an OGTT was associated with increased identification of women with GDM and severe hyperglycaemia and with neonatal benefits for those with GDM. There was no evidence of clear differences in neonatal outcomes in the whole obstetric population, which is perhaps not surprising as only 6% were identified as having GDM when an OGTT was offered to all women and GDM diagnosis made based on the WHO criteria.

### Ethics

Ethical approval was not required for this work.

## Electronic supplementary material

Additional file 1: Proportions of pregnant women who completed an OGTT who had documented risk factors* for GDM. (DOC 32 KB)

## Abbreviations

GDM: Gestational diabetes mellitus
OGTT: Oral glucose tolerance test
NNU: Neonatal unit
BMI: Body mass index
WHO: World Health Organisation (WHO)
ACOG: The American College of Obstetricians and Gynecologists
UK: United Kingdom
NICE: National Institute for Health and Care Excellence.
