# Supplementary material for: Evaluation of the impact of universal testing for gestational diabetes mellitus on maternal and neonatal health outcomes: a retrospective analysis
Source: BMC Pregnancy Childbirth. 2014 Sep 9;14:317. doi: 10.1186/1471-2393-14-317 (PMC4167281; doi:10.1186/1471-2393-14-317)
Supplement: Supplementary file 1 — Additional file 1: Proportions of pregnant women who completed an OGTT who had documented risk factors* for GDM. (DOC 32 KB) [file 12884_2014_1184_MOESM1_ESM.doc]

| Additional file 1: Proportions of pregnant women who completed an OGTT who had documented risk factors* for GDM |
| --- |
|  |
| |  | **Number (%) [95% CI] with each risk factor amongst women completing an OGTT** | | | --- | --- | --- | |  | Completed an OGTT during the selective  offer period (2004-2006)  N = 1151 | Completed an OGTT during the universal offer period (2008-2010)  N = 11,516 | | Previous gestational diabetes | 124 (11.7) [9.8-13.6] | NA | | Previous delivery of infant with macrosomia | 78 (7.4) [5.8-9.0] | NA | | Family history of diabetes | 191 (18.1) [15.8-20.4] | NA | | Obesity (BMI > 30 kg/m^2^) | 252 (23.9) [21.3-26.4] | NA | | From an at-risk ethnic background^a^ | 610 (57.8) [54.8-60.7] | 6491 (55.4) [54.5-56.3] | | Other risk factor^b^ | 295 (27.9) [25.2-30.6] | NA | |

*Women may have multiple risk factors

CI = confidence interval; OGTT =oral glucose tolerance test; BMI = body mass index; NA = not available

^a^South Asian, black or middle eastern ethnicity

^b^glycosuria, raised random blood glucose, increased liquor volume
